# Usability and Teachability of Continuous Glucose Monitoring Devices in Older Adults and Diabetes Educators: Task Analysis and Ease-of-Use Survey

**DOI:** 10.2196/42057

**Published:** 2022-12-15

**Authors:** Simon Psavko, Noam Katz, Tina Mirchi, Courtney R Green

**Affiliations:** 1 Dexcom, Inc San Diego, CA United States

**Keywords:** medical devices, wearable devices, older adults, task analysis, usability testing, continuous glucose monitoring, glucose monitoring, glucose levels, diabetes, usability

## Abstract

**Background:**

Continuous glucose monitoring (CGM) devices continuously sense and relay glucose concentration data from the interstitial fluid to a mobile phone or receiver. Older adults benefit from this continuous monitoring of glucose levels. Proper deployment of the sensing wire is facilitated by a specialized applicator.

**Objective:**

Our aim was to assess a new seventh-generation (G7) CGM device (Dexcom, Inc) for use by adults 65 years of age or older and certified diabetes care and education specialists (CDCESs). Ease of use related to intradermal insertion and mobile app setup will be assessed and compared to the fifth- and sixth-generation systems.

**Methods:**

Formal task analysis was conducted to enumerate the number and complexity of tasks associated with CGM deployment. We recruited 10 older adults with no prior CGM experience and 10 CDCESs to assess ease of use through hands-on insertion and initiation of a G7 system followed by a survey and, for older adults, a system usability scale survey.

**Results:**

About half as many tasks are needed to deploy G7 compared to G6. Older adults and CDCESs reported overall high usability of the G7 CGM device. CDCESs noted G7’s easier setup compared to previous generations. The system usability scale score for the CGM system was 92.8, which reflects excellent usability.

**Conclusions:**

For CDCESs and for older adults using the G7 CGM system, cognitive burden is relatively low and reduced compared to previous CGM systems. Easing of this burden and simplification of the glucose monitoring aspect of proper diabetes management will likely contribute to improved outcomes in this population.

## Introduction

A substantial proportion of people with diabetes do not reach the goals of treatment [1], which is due in part to barriers to effective self-care. Real-time continuous glucose monitoring (RT-CGM) is recommended for patients with diabetes using insulin [2], and many studies support the utility of continuous glucose monitoring (CGM) devices with respect to reducing HbA1c and improving outcomes [3,4]. Ease of use (objectively or subjectively determined) is critically important because this is a key factor in CGM compliance and favorable clinical outcomes.

Certified diabetes care and education specialists (CDCESs) are health care professionals with expertise in diabetes prevention, prediabetes, and diabetes management. They often work as part of a care team to help those with diabetes understand their disease and achieve their goals in managing diabetes. A recent study found that glycemic and metabolic outcomes were similar in patients who received a telehealth-based diabetes consultation with an endocrinologist or a diabetes self-management education visit with a CDCES [5]. Because CDCESs train patients who are new to CGM, their comfort with CGM device insertion and app setup is also critical to patient training and adoption.

Management of diabetes has evolved dramatically [6], and CGM use is rapidly proliferating. Still, diabetes self-management imposes cognitive demands and requires a significant level of expertise [7]. Recent studies demonstrated an improvement in glycemic outcomes with CGM for patients 65 years or older [8] that was sustained for an additional 12 months [9]. Although usage in older adults was very high in these studies (at least 83% of participants wore a RT-CGM device ≥6 days/week over the 6-month study period [8]), barriers still exist to broad population-level use. Wildenbos et al [10] categorized these barriers as related to cognition, motivation, physical ability, and perception. This can include specific barriers such as memory decline, arthritis (especially in the hands), impaired vision or hearing, and perception of the technology as too complex [10,11]. These barriers can be lowered through enhanced usability of CGM. RT-CGM has the potential to improve quality of life in older adults, but adoption barriers must be considered and addressed [11].

A seventh-generation CGM system (G7; Dexcom, Inc) received CE mark in March 2022 and provides accurate estimates of glucose levels in the interstitial fluid in adults [12] as well as children and adolescents [13]. Clinical benefits are anticipated due to the similarity of accuracy metrics between the fifth-, sixth-, and seventh-generation systems and an increased feature set [14].

To maximize usability, G7 was designed using human factors engineering and usability engineering processes. Human factors analysis throughout product development has been shown to enhance usability of CGM sensor applicators [15]. In designing the device, participants representative of the intended user population were evaluated on their ability to use the CGM system safely and effectively. This included 3 human factors experts who performed multiple heuristic reviews of the device and 381 participants representative of the intended user population who participated across 26 usability studies, evaluating all aspects of the Dexcom G7 system hardware, software, and labeling. All usability studies were conducted in accordance with US Food and Drug Administration and international guidance [16-19].

There is a paucity of CGM usability studies specifically in special populations such as older adults and diabetes educators. In this study, we describe a task analysis and ease-of-use study conducted with a commercially representative G7 system with 10 CDCESs and 10 CGM-naïve older adults with type 2 diabetes (T2D). Relevant surveys were conducted of each group, and responses to open-ended discussions were recorded.

## Methods

### Task Analysis

A task analysis and heuristic evaluation was conducted on the fifth-, sixth-, and seventh-generation CGM systems. The task analysis identified the perceptual inputs, cognitive processes, and actions required for a user to complete the task of sensor insertion. The heuristic evaluation was based on existing design principles and compliance with recognized usability guidelines to uncover potential use errors and end user risks. The number of tasks required for sensor insertion was enumerated as well as the potential unrecoverable use errors, which are defined as incorrect actions committed by the user during the sensor insertion process that would result in predeployment loss of the device. Potential errors that would lead to sensor loss include failing to remove one or both of the adhesive backings from the applicator prior to deploying the sensor and sensor deployment before proper placement of the device on the skin.

### Usability Study

Although it is anticipated that CGM-naïve patients initiating CGM use would receive one-on-one training, discuss personal-use CGM**,** and be directed to additional web-based training prior to using CGM, this study chose to evaluate a ‘no-training’ scenario for the following 3 reasons: (1) it is difficult to provide an equivalent level of detail across different instances of one-on-one training; (2) one-on-one training may not occur in every real-world instance; and (3) lack of training represents the most stringent evaluation of safe and effective usability.

In an in-person, one-on-one setting, 10 CDCESs and 10 CGM-naïve older adults with T2D were provided with a G7 system and a mobile phone app. Both groups were tasked with setting up the mobile app, deploying the sensor on themselves, and establishing communication between the app and the sensor without training or instructions beyond what is included in the G7 box and mobile app. This included allowing all notifications, pairing the wearable, reviewing safety information, reading the alert functionality explanation, watching the required videos, and following sensor insertion instructions. During the study session, all relevant activities performed by participants, including successful and unsuccessful task completion, root causes, and salient participant questions or comments, were recorded. Their total sensor insertion time was also recorded. Following the initiation of G7, both groups completed a posttest survey. Responses to open-ended questions were also recorded. The older adult cohort also completed a system usability scale (SUS) survey [20] to assess their comfort with CGM hardware and app setup as CGM-naïve users. Both surveys (ie, posttest and SUS) evaluated responses using a 5-point Likert scale (1=strongly disagree and 5=strongly agree).

### Ethical Considerations

The G7 CGM device had CE marking at the time of the study. This ease-of-use study was not part of an intervention or trial, and the device was not used to make diagnostic or treatment decisions. Similar to the submission of human factors reports to the Food and Drug Administration, which does not require an institutional review board or independent ethics committee approval [16], these approvals were not sought for this ease-of-use study.

## Results

### Task Analysis Found Fewer Tasks Required for G7 Insertion

**Figure 1:**
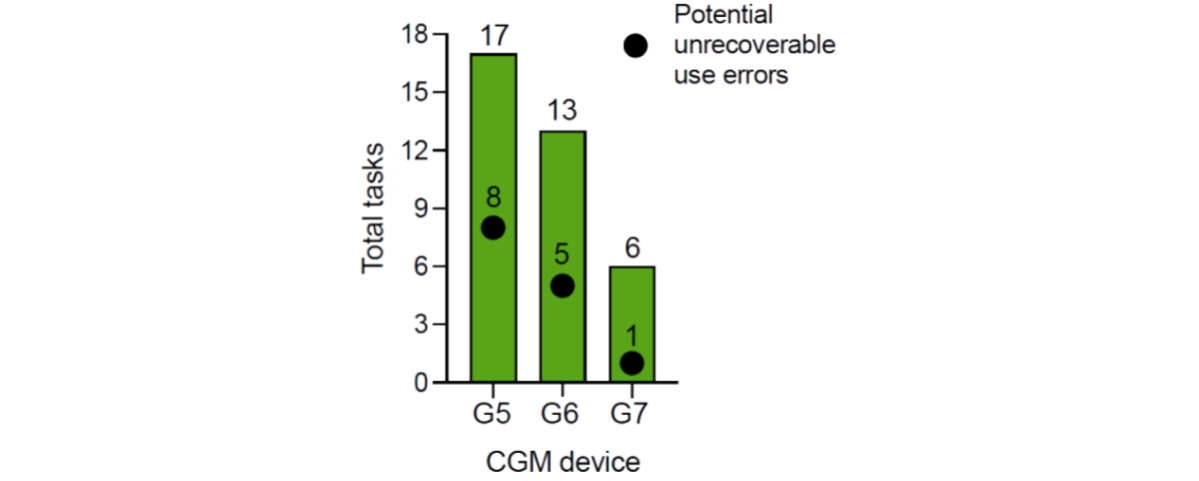
Summary of tasks required for each generation (fifth generation: G5; sixth generation: G6; seventh generation: G7) of Dexcom's continuous glucose monitoring (CGM) devices.

Insertion and initialization of the G7 system was subjected to a task analysis. The number of user tasks required for sensor insertion decreased with each subsequent generation (G5: 17 tasks; G6: 13 tasks; and G7: 6 tasks; Figure 1). In addition, the number of potential unrecoverable use errors also decreased (G5: 8; G6: 5; and G7: 1; Figure 1). The full task analyses of each device are summarized in Tables S1-S3 in Multimedia Appendix 1.

Two groups were recruited to participate in this study. A total of 10 CDCESs with an average of 15.7 years of experience agreed to participate. On average, CDCESs train 18 patients on CGM use per month, and a majority of their patients use CGM (Table 1). The second group consisted of 10 older adults with T2D. Their average age was 69.7 years, all were CGM-naïve, and all were on a multiple daily injection insulin regimen (Table 1).

**Table 1:** Demographics of certified diabetes care and education specialists and older adult participants.

Participants		Values
**Certified diabetes care and education specialists (N=10)**		
	Years of experience, mean (range)	15.7 (5-25)
	Patients trained on CGM^a^ per month, mean (range)	18 (5-50)
	Patients using CGM (%), mean	58.5
**Older adults with type 2 diabetes (N=10)**		
	Age, mean (range)	69.7 (65-79)
	CGM-naïve, n (%)	10 (100)
	Treatment by multiple daily insulin injections, n (%)	10 (100)

^a^CGM: continuous glucose monitoring.

### CDCESs Usability Ratings

All CDCES participants successfully completed G7 app setup and sensor insertion. The time on task was recorded from the start of app onboarding setup through completion of the onboarding (including sensor insertion). The total setup time ranged from 6 minutes to 9.2 minutes (average=7.15 minutes). In the CDCES posttest survey, average ratings were very favorable, and only one participant provided a rating that was assessed to be less than neutral (Table 2). In particular, the statements “the system was easy to learn to use,” “I believe this system is easy to train patients to use,” and “I believe this system is easy to set up” received mean ratings of 5.0 (“strongly agree”) out of a possible score of 5.

In the open-ended discussion, CDCESs were asked to discuss their experience with G7 compared to other CGM devices they use. The participants expressed that setting up and initiating the seventh-generation CGM is extremely easy, requires fewer steps, and feels much less confusing compared to other products. CDCES participants were also asked to discuss their views of virtual CGM training. These participants viewed virtual training of the CGM system as practical and believed it would be very easy for patients to do the training at home on their own.

**Table 2:** Posttest survey results from interviews with 10 certified diabetes care and education specialists on use of the seventh-generation (G7) continuous glucose monitoring system.

Statement	Mean	Range	Rating and rationale for ratings lower than neutral
I could effectively complete the tasks that were given to me.	4.9	4-5	N/A^a^
I felt comfortable using this system.	4.9	4-5	N/A
The system was easy to learn to use.	5.0	5	N/A
Whenever I made a mistake, I could recover easily and quickly.	4.7	3-5	Rating of 3: participants did not make any mistakes and thus rated this statement as neutral.
The functions I saw worked as I would expect.	4.6	2-5	Rating of 2: participant stated the sound of the sensor insertion deployment was very loud.
The system showed information clearly and effectively.	4.8	4-5	N/A
I found this system unnecessarily complex.	1.0	1	N/A
I believe this system is easy to train patients to use.	5.0	5	N/A
I believe this system is easy to set up.	5.0	5	N/A
I believe a patient can self-train on this system.	4.8	4-5	N/A
It is easier to train on G7 than G6.	4.9	4-5	N/A
G7 setup requires less time to train or set up than G6.	4.9	4-5	N/A

^a^N/A: not applicable.

### Older Adults With T2D Usability Ratings

All CGM-naïve older adult participants successfully completed G7 app setup and sensor insertion. The time on task was recorded from the start of the app setup through onboarding completion (including sensor insertion), with setup time ranging from 9 minutes to 18 minutes (average=12.6 minutes). Sensor insertion time was also recorded, with insertion time ranging from 58 seconds to 3 minutes (average=1.95 minutes).

A posttest survey and a SUS survey were given to the older adult participants (Table 3 and Table S4 in Multimedia Appendix 2). No responses lower than neutral were recorded in the posttest survey (Table 3). The SUS score for setup and insertion of the G7 system was 92.8 (Table S4 in Multimedia Appendix 2). This score reflects an excellent usability rating [21].

In the open-ended discussion, older adults were also asked to discuss how comfortable they would be setting up G7 on their own with no training or assistance. All participants stated they would feel “comfortable” or “very comfortable” and specified that the app setup and insertion instructions were concise and easy to follow. They stated that the instructions, images, and in-app videos during setup are helpful. Overall, the G7 system provides increased usability, when compared to previous generations, in terms of efficacy, efficiency, ease of user learning, and user satisfaction.

**Table 3:** Posttest survey results from interviews with 10 older adults with type 2 diabetes on use of the seventh-generation (G7) continuous glucose monitoring system.

Statement	Mean	Range	Rating and rationale for ratings lower than neutral
I could effectively complete the tasks that were given to me.	4.9	4-5	N/A^a^
I felt comfortable using the G7 system.	4.8	4-5	N/A
The G7 system was easy to learn to use.	4.7	4-5	N/A
The functions I saw worked as I would expect.	4.7	3-5	N/A
The G7 system showed information clearly and effectively.	4.9	4-5	N/A
I believe the G7 system is easy to set up.	4.6	3-5	N/A
I believe I can set up the G7 system on my own.	4.9	4-5	N/A
I believe I can set up the G7 system in a virtual training.	4.8	4-5	N/A

^a^N/A: not applicable.

## Discussion

The analyses presented in this paper constitute the first ease of use study of the Dexcom G7 RT-CGM. Previously published results of a task analysis [14] and various survey results presented in this study show that the G7 sensor is easier to insert and set up compared to the two previous device generations. The reduction in potential unrecoverable use errors also reduces the likelihood of wasting a sensor if a mistake is made during the setup or insertion process. Simpler sensor insertion and app setup processes allow the seventh-generation CGM system to be even easier for older adults to learn and use, which may aid in their motivation to try a new technology [11]. RT-CGM with its alerts and alarms has been shown to contribute to better glycemic outcomes in older adults, including severe hypoglycemia (SH), which is particularly dangerous in this population [8,9]. In a study by the Wireless Innovation for Seniors With Diabetes Mellitus study group [8], CGM users achieved an adjusted difference of –1.9 percentage points in time below 70mg/dL compared to the standard blood glucose monitoring group. This benefit was sustained after 12 months of CGM use [9].

Extensive design work and human factors analyses contributed to the design of the seventh-generation system, and a multifaceted ease-of-use study was described in this paper. This study included an objective task analysis and surveys of CDCESs and older adults with diabetes, which demonstrated robust improvements in the ease of use and overall comfort with the device. A similar study assessed the ease-of-use ratings of the Dexcom G6’s automatic sensor applicator versus the Dexcom G5’s manual sensor applicator [15]. In our study, we interviewed both older adults with T2D and CDCESs because although people with diabetes are the end users of the device, CDCESs are the primary individuals who would train and educate older adults, and therefore, their comfort with device operation is critical.

CDCESs work on a diabetes care team to develop strategies with patients for successful diabetes management. They play a unique role in helping patients understand their diabetes and learn new technologies [22,23]. Highly CGM-literate CDCESs could aid in CGM adoption by older adults. A recent study of patients with type 1 diabetes covered by Medicare found that CGM adoption remains low, and disparities persist between racial groups [24]. Improved patient education and exposure could reduce these disparities. In a recent study of 171 users of a blood glucose monitoring device with T2D, 29/171 (21%) expressed a lack of interest in CGM adoption [25]; 4/29 (14%) respondents cited a lack of familiarity with CGM as the reason for their disinterest [25]. Recently, more patients gained access to CGM when the Centers for Medicare and Medicaid Services expanded coverage to any CGM that connects to an insulin pump or a standalone receiver and eliminated the requirement for 4 finger sticks per day [26,27]. A proposed local coverage determination from Centers for Medicare and Medicaid Services also would expand CGM coverage to those with a history of problematic hypoglycemia, regardless of insulin use [28]. This is encouraging because the association between CGM use and improved outcomes in people with T2D is strong and growing [3,29,30].

Adoption of CGM in older adults is particularly important for several reasons. The most important aspect of glucose management in older adults is the avoidance of SH. Older patients are more likely to have hypoglycemia unawareness, a reduced ability to produce counterregulatory hormones, and an altered metabolism that increases the risk of SH due to polypharmacy [11]. The risks associated with SH include falls leading to fractures or other injuries, cardiovascular complications, and temporary or permanent cognitive impairment [11,31,32]. Because of the high risk associated with SH in older adults, an International Consensus on Time in Range recommended a more relaxed set of CGM-based targets with the goal of avoiding hypoglycemia [33]. If needed, coordination with a CDCES or other health care providers to adjust alert thresholds and settings could improve user experience.

This study contained some limitations. The small sample size, inclusion of only US participants, and inclusion of only CDCESs (ie, a highly skilled but small group of health care providers) limit the generalizability of the results. This study also did not assess long-term use of the G7 and, because no data were generated, usability of the app interface was not tested.

Ease of use is a critically important element in all diabetes technology, including CGMs. This seventh-generation device was designed using human factors engineering and usability engineering processes, and iterations were performed after reviews from hundreds of potential users. Increased adoption of these technologies relies on appropriate usability, safety, effectiveness, and robust design [34,35]. Older adults report excellent G7 usability and CDCESs report that the G7 is simpler and easier to use compared to the two previous generations of the system. Data presented in this study should inform prescribing and treatment decisions in this vulnerable population.

## Appendix

Multimedia Appendix 1 Full task analyses of the fifth-, sixth-, and seventh-generation real-time continuous glucose monitoring devices.

Multimedia Appendix 2 Posttest system usability scale (SUS) results from interviews with 10 older adult participants.

## Abbreviations

CDCES: certified diabetes care and education specialist
CGM: continuous glucose monitoring
RT-CGM: real-time continuous glucose monitoring
SH: severe hypoglycemia
SUS: System Usability Scale
T2D: type 2 diabetes
