# Supplementary material for: Usability and Teachability of Continuous Glucose Monitoring Devices in Older Adults and Diabetes Educators: Task Analysis and Ease-of-Use Survey
Source: JMIR Hum Factors. 2022 Dec 15;9(4):e42057. doi: 10.2196/42057 (PMC9801269; doi:10.2196/42057)
Supplement: Multimedia Appendix 1 [file humanfactors_v9i4e42057_app1.docx]

**Supplementary Table 1.** Task Analysis of G7 insertion

| **G7 Task 1: Insert G7 sensor into the body** | | |
| --- | --- | --- |
| **Step** | **Task Name** | **Task Behavior: Indicator (I), Decision (D),**  **Action (A), Feedback (F)** |
| 1.1 | Identify components: applicator | (I) Components have not been identified  (D) Need to open applicator box to reveal applicator  (A) Open applicator box  (F) Applicator is revealed |
| 1.2 | Unscrew cap from applicator | (I) Cap is on applicator  (D) Need to remove cap from applicator to reveal sensor  (A) Twist cap to remove  (F) Cap is detached from applicator and sensor patch is revealed |
| 1.3 | Place applicator firmly against skin | (I) Applicator has no cap and is not touching skin  (D) Need to place applicator firmly on skin so that the sensor can be deployed  (A) Place applicator on skin and press firmly until the clear safety guard can’t be seen  (F) Clear safety guard is hidden from view |
| 1.4 | Press button on applicator to deploy sensor | (I) Button has not been pressed and sensor is not deployed  (D) Need to press button to deploy sensor  (A) Press button while clear safety guard is pushed in  (F) Button depresses, an audible click sounds, sensor is deployed |
| 1.5 | Remove applicator from skin | (I) Applicator is touching skin  (D) Need to remove the applicator from skin to reveal the sensor  (A) Applicator is pulled away from skin  (F) Sensor with patch is seen on skin |
| 1.6 | Enter pairing code into display device | (I) Pairing code has not been entered into display device  (D) Need to enter pairing code into display device for communication with sensor  (A) Pairing code is entered into display device  (F) Sensor is paired to display device |

**Supplementary Table 2.** Task Analysis of G6 insertion

| **G6 Application, Task 1: Insert G6 sensor into the body** | | |
| --- | --- | --- |
| **Step** | **Task Name** | **Task Behavior: Indicator (I), Decision (D),**  **Action (A), Feedback (F)** |
| 1.1 | Identify components: applicator | (I) Applicator has not been identified  (D) Need to open applicator box to reveal applicator  (A) Open applicator box  (F) Applicator is revealed |
| 1.2 | Identify components: transmitter | (I) Transmitter has not been identified  (D) Need to open transmitter box to reveal transmitter  (A) Open transmitter box  (F) Transmitter is revealed |
| 1.3 | Remove applicator from thermoform | (I) Applicator is in thermoform packaging  (D) Need to remove applicator from thermoform packaging to use  (A) Thermoform packaging is opened  (F) Applicator is unobstructed |
| 1.4 | Remove transmitter from transmitter box | (I) Transmitter is in transmitter box  (D) Need to remove transmitter from transmitter box to use  (A) Pull transmitter out of transmitter box  (F) Transmitter is not in transmitter box |
| 1.5 | Enter sensor code into display device | (I) Sensor code has not been entered into display device  (D) Need to enter sensor code into display device for communication with sensor  (A) Sensor code is entered into display device  (F) Sensor is registered to display device |
| 1.6 | Enter transmitter code into display device | (I) Transmitter code has not been entered into display device  (D) Need to enter transmitter code into display device for communication with transmitter  (A) Transmitter code is entered into display device  (F) Transmitter is paired to display device |
| 1.7 | Remove adhesive backings | (I) Adhesive backings are attached to sensor adhesive and contain pull tabs  (D) Need to remove backings to expose sensor adhesive  (A) Pull tabs to remove backings  (F) Backings peel away from sensor adhesive |
| 1.8 | Orient and place applicator on skin | (I) Adhesive is exposed  (D) Need to place applicator on skin to insert sensor  (A) Place exposed applicator adhesive on skin  (F) Applicator adhesive is touching skin |
| 1.9 | Fold and remove safety guard | (I) Safety guard is covering applicator button  (D) Need to remove safety guard to press button  (A) Fold and break off safety guard  (F) Safety guard is not attached to applicator |
| 1.10 | Press button on applicator to deploy sensor | (I) Button is unobstructed by safety guard  (D) Need to press button to deploy sensor  (A) Press button down with finger  (F) Button is recessed, an audible click sounds, sensor is deployed from applicator |
| 1.11 | Remove applicator from skin | (I) Applicator is touching skin  (D) Need to remove the applicator from skin to reveal the transmitter holder  (A) Applicator is pulled away from skin  (F) Transmitter holder and patch is seen on skin |
| **G6 Application, Task 2: Attach transmitter to the holder** | | |
| **Step** | **Task Name** | **Task Behavior: Indicator (I), Decision (D),**  **Action (A), Feedback (F)** |
| 2.1 | Insert transmitter into holder | (I) Transmitter is not in holder  (D) Need to place transmitter into holder tab first  (A) Transmitter is placed into holder tab first in correct orientation  (F) Entire transmitter fits into holder |
| 2.2 | Snap transmitter into place | (I) Transmitter is not flat and snug in holder  (D) Need to snap transmitter into holder to complete G6 insertion  (A) Transmitter is pressed down into holder  (F) Transmitter is flat and flush with holder with an audible click sound |

**Supplementary Table 3.** Task analysis of G5 insertion

| **G5 Task 1: Insert G5 sensor into the body** | | |
| --- | --- | --- |
| **Step** | **Task Name** | **Task Behavior: Indicator (I), Decision (D),**  **Action (A), (F) Feedback** |
| 1.1 | Identify components: applicator | (I) Applicator has not been identified  (D) Need to open applicator box to reveal applicator  (A) Open applicator box  (F) Applicator is revealed |
| 1.2 | Identify components: transmitter | (I) Transmitter has not been identified  (D) Need to open transmitter box to reveal transmitter  (A) Open transmitter box  (F) Transmitter is revealed |
| 1.3 | Remove applicator from packaging | (I) Applicator is in packaging  (D) Need to remove applicator from packaging to use  (A) packaging is opened  (F) Applicator is unobstructed |
| 1.4 | Remove transmitter from transmitter box | (I) Transmitter is in transmitter box  (D) Need to remove transmitter from transmitter box to use  (A) Pull transmitter out of transmitter box  (F) Transmitter is not in transmitter box |
| 1.5 | Enter transmitter code into display device | (I) Transmitter code has not been entered into display device  (D) Need to enter transmitter code into display device for communication with transmitter  (A) Transmitter code is entered into display device  (F) Transmitter is paired to display device |
| 1.6 | Remove adhesive backings | (I) Adhesive backings are attached to sensor adhesive and contain pull tabs  (D) Need to remove backings to expose sensor adhesive  (A) Pull tabs to remove backings  (F) Backings peel away from sensor adhesive |
| 1.7 | Orient and place applicator on skin | (I) Adhesive is exposed  (D) Need to place applicator on skin to insert sensor  (A) Place exposed applicator adhesive on skin  (F) Applicator adhesive is touching skin |
| 1.8 | Smooth out adhesive on skin | (I) Adhesive is folded upwards and not flush against site  (D) Need to ensure adhesive is flush against body and sticks  (A) Smooth out adhesive  (F) Adhesive is flush against skin |
| 1.9 | Pull out safety lock | (I) Safety lock is preventing applicator from being pressed down  (D) Need to remove safety lock to depress applicator  (A) Pull safety lock straight out  (F) Safety lock is not attached to applicator |
| 1.10 | Pinch up subcutaneous tissue | (I) Safety lock is preventing applicator from being pressed down  (D) Need to insert into fatty tissue  (A) Pinch up on skin  (F) Subcutaneous tissue is pinched, and fatty tissue area increased |
| 1.11 | Position fingers and hands for insertion | (I) Subcutaneous tissue is pinched  (D) Need to grasp applicator  (A) Place fingers above the collar and thumb on plunger  (F) Thumb is on plunger and applicator feels secure |
| 1.12 | Press down on plunger to deploy sensor | (I) Plunger is unobstructed  (D) Need to press plunger to deploy sensor  (A) Press plunger down with thumb  (F) Button is recessed, an audible click sounds, sensor is deployed from applicator |
| 1.13 | Remove introducer needle | (I) Applicator is touching skin  (D) Need to remove the applicator from skin to reveal the transmitter holder  (A) Pull up on collar until resistance  (F) Cannot pull on collar any further |
| 1.14 | Remove applicator from transmitter holder | (I) Applicator is attached to transmitter holder  (D) Need to remove the applicator from transmitter holder  (A) Squeeze tabs on side of transmitter holder and move applicator forward  (F) Transmitter holder and patch is seen on skin |
| **G5 Task 2: Attach transmitter to the holder** | | |
| **Step** | **Task Name** | **Task Behavior: Indicator (I), Decision (D),**  **Action (A), Feedback (F)** |
| 2.1 | Insert transmitter into holder | (I) Transmitter is not in holder  (D) Need to place transmitter into holder tab first  (A) Transmitter is placed into holder tab first in correct orientation  (F) Entire transmitter fits into holder |
| 2.2 | Snap transmitter into place | (I) Transmitter is not flat and snug in holder, and transmitter latch is connected  (D) Need to snap transmitter into holder to complete G5 insertion  (A) Transmitter is pressed down into holder, and transmitter latch pulled up and forward  (F) Transmitter is flat and flush with holder with an audible click sound |
| 2.3 | Remove transmitter latch | (I) Transmitter latch is connected to holder  (D) Need to remove transmitter latch  (A) Twist off transmitter latch away from body  (F) Transmitter latch is no longer connected to transmitter holder |
