# Supplementary material for: Usability and Teachability of Continuous Glucose Monitoring Devices in Older Adults and Diabetes Educators: Task Analysis and Ease-of-Use Survey
Source: JMIR Hum Factors. 2022 Dec 15;9(4):e42057. doi: 10.2196/42057 (PMC9801269; doi:10.2196/42057)
Supplement: Multimedia Appendix 2 [file humanfactors_v9i4e42057_app2.docx]

**Supplementary Table 4.** Post-test System Usability Scale (SUS) results from interviews with 10 older adult participants

| **Statement** | **Mean** | **Range** | **Rationale for Ratings Lower than Neutral** |
| --- | --- | --- | --- |
| 1. I think that I would like to use the G7 system. | 4.9 | 4-5 | N/A |
| 1. I found the G7 system unnecessarily complex. | 1.3 | 1-3 | N/A |
| 1. I thought the G7 system was easy to use. | 4.9 | 4-5 | N/A |
| 1. I think that I would need the support of a technical person to be able to use the G7 system. | 1.6 | 1-4 | **Rating of “4”:** Participant stated they are unfamiliar with smartphones and currently use a flip phone. |
| 1. I found the various functions were well integrated. | 4.8 | 4-5 | N/A |
| 1. I thought there was too much inconsistency in the G7 system. | 1.2 | 1-3 | **Rating of “3”:** Participant stated they sometimes still need to use their blood glucose meter and thus rated this statement as neutral |
| 1. I would imagine that most people would learn to use the G7 system very quickly. | 4.5 | 3-5 | N/A |
| 1. I found the G7 system very awkward to use. | 1.4 | 1-4 | N/A |
| 1. I felt very confident using the G7 system. | 4.9 | 4-5 | N/A |
| 1. I needed to learn a lot of things before I could get going with the G7 system. | 1.4 | 1-4 | **Rating of “4”:** Participant stated they are unfamiliar with smartphones and currently use a flip phone. |
